# A new case of primary signet-ring cell carcinoma of the cervix with prominent endometrial and myometrial involvement: Immunohistochemical and molecular studies and review of the literature

**DOI:** 10.1186/1477-7819-10-7

**Published:** 2012-01-11

**Authors:** Giovanna Giordano, Silvia Pizzi, Roberto Berretta, Tiziana D'Adda

**Affiliations:** 1 Department of Pathology and Medicine of Laboratory, Section of Pathology, Parma University; 2 Department of Obstetric and Gynaecologic Sciences and Neonatology, Parma University

**Keywords:** Signet-ring cell carcinoma, Cervical adenocarcinoma, Human Papilloma Virus (HPV), Polymerase Chain Reaction Amplification (PCR)

## Abstract

**Background:**

As a rule, endocervical tumours with signet-ring cell are classed as metastatic extra-genital neoplasms. In a patient aged 45 years, we describe primary cervical signet-ring cell carcinoma (PCSRCC) characterized by prominent endometrial and myometrial involvement, simulating primary endometrial adenocarcinoma with cervical extension. In addition, a review was made of the literature to identify the clinical and pathological features of this rare malignancy.

**Case presentation:**

A 45-year-old woman was referred to our Gynaecology Department due to persistent abnormal vaginal bleeding. Transvaginal ultrasonography showed slight endometrial irregularities in the whole uterine cavity suggestive of endometrial neoplasms. Pelvic magnetic resonance imaging revealed diffuse enlargement of the cervix, which had been replaced by a mass. Induration extended to the parametria and sigmoid colon fat.

Histological examination of endometrial curettage and a cervical biopsy revealed a neoplasm characterized by neoplastic signet-ring cells and trabecular structures. Immunohistochemical analysis and molecular studies showed certain findings consistent with a cervical neoplasm, such as positivity to CEA, keratin 7, Ca-125 and p16 and the presence of HPV (Human Papilloma Virus) DNA 18.

On examination of the hysterectomy with bilateral salpingo-oophorectomy and pelvic lymphadenectomy, the lesion replacing the cervix, endometrium and myometrium, revealed the same immunohistochemical findings observed on endometrial curettage and cervical biopsy specimens. Metastases were found in an ovarian cystic lesion and the lymph nodes.

**Conclusion:**

With this report the authors have demonstrated that the spread of cervical adenocarcinoma to the uterine corpus, although rare, may be observed, and that in this instance immunohistochemical and molecular studies can provide sufficient information for accurate diagnosis even on small biopsy specimens.

## Background

Signet-ring cell carcinoma is an epithelial malignancy characterized by the presence of cells with a signet-ring appearance. This appearance is due to a large vacuole full of mucin displacing the nucleus to the periphery. Thus, the pool of mucin in a signet-ring cell mimics the appearance of a finger hole, while the nucleus represents the face of the ring in profile [1]. Signet-ring cell carcinoma is considered a form of adenocarcinoma [1].

Signet-ring cells are most frequently associated with stomach cancer [1], but may be observed in any tissue including the prostate [2] bladder, gallbladder [3] breast, and colon [4] as well as in stromal tumours of the ovary and testis [5].

Most signet-ring cell carcinomas in the female genital tract are extra-genital metastatic neoplasms [3,6-9].

Primary signet-ring carcinomas of the female genital organs, on the other hand, may be considered a rare event. In fact, primary signet-ring cell carcinoma of the endometrium has only been observed in 4 previous cases [10-12].

Moreover, to the best of our knowledge, no more than 14 cases of primary cervical carcinoma containing signet-ring cells have been reported in the English literature [13-22].

In this paper, we report a new case of primary signet-ring cell carcinoma of the cervix (PSRCC) with prominent endometrial and myometrial involvement simulating endometrial adenocarcinoma with extension to the cervix or metastatic extra-genital tumour (OK). The results of immunohistochemical and molecular studies have been reported as ancillary techniques to provide information for a more accurate diagnosis of this rare malignancy. In addition, we reviewed the literature to identify the clinical and pathological features of this rare malignancy.

## Case presentation

The materials consisted of cervical and endometrial biopsy specimens, plus specimens from hysterectomy with bilateral salpingo-oophorectomy and pelvic lymphadenectomy. The specimens were fixed in 10% neutral-buffered formalin for a routine light microscope examination. The samples were embedded in paraffin, then 3μ sections were cut and stained with haematoxylin-eosin. Immunohistochemistry was performed with antibodies anti-Ca 125 (Dilution 1:100, clone: M11, Dako, Glostrup, Denmark), CEA (Dilution 1:200, clone: 11-7, Dako, Glostrup, Denmark), anti keratin 7 (Dilution:1:200, clone OV-TL 12/30, Neomarkers, Pleasalton-CA), and p16^INK4a ^(Kit Cinetec p16 ^INK4a^, Heidelberg, Germany), Vimentin (Neomarkers, dilution:1:500, clone:V9, Neomarkers, Pleasalton-CA), anti-oestrogen receptors (Dilution:1:50, clone: SP1, Neomarkers, Pleasalton-CA), anti-progesterone receptors, (Dilution:1:200, clone: P8R636, Dako, Glostrup, Denmark), Chromogranin A **(**Dilution:1:2000, clone: LK 2H10+ PHE5, Neomarkers, Fremont-CA) and Synaptophysin **(**Dilution:1:100, polyclonal, Cell Marque, Hot Springs, AR, USA), using the avidin-biotin method.

Polymerase chain reaction amplification (PCR) was performed to evaluate the presence of HPV DNA in the cervical and endomyometrial neoplasm.

For DNA extraction, 4 μm-thick histological sections were stained with haematoxylin and examined under a stereomicroscope. Neoplastic areas were manually micro-dissected using sterile scalpels, suspended in a buffer for tissue lysis (Tris HCl 50 mM, pH 9, 1 mM EDTA pH 8.0, 0.5% Tween 20, 5% Chelex 100), and incubated overnight with Proteinase K (0.4 mg/ml) at 55°C. After enzyme inactivation by 10 min boiling, DNA extracted was used directly in the PCR mix, without further purification.

HPV-PCR amplification was performed with L1 consensus primers Gp5+/Gp6+ [23], giving an expected PCR product size of 150 bp: these primers, which were distinct but pooled, have been developed to allow the detection of a broad spectrum of mucosotropic HPV genotypes (6, 11, 13, 16, 18, 30-35, 39, 40, 42, 45, 51-53, 56, 58, 61, 66). Most of these genotypes are correlated with lesions of high oncogenic risk (16,18,45,56 and 58). Five μl of appropriately diluted DNA were combined in a 25 μl reaction mixture containing 10 mM Tris-HCl (pH 9.0), 50 mM KCl, 0.1% Triton X-100, 200 μM of each dNTP (Promega, Madison, WI, USA), 0.4 μM of each primer, 2.0 mM MgCl_2_, 1.25 U Taq polymerase (Promega, Madison, WI, USA). Amplification was carried out for 40 cycles in an AB 2700 (Applied Biosystems, Foster City, CA, USA) thermal cycler. Each cycle of amplification consisted of 1 min denaturation at 94°C, 1 min annealing at 46°C, and 1 min of elongation at 72°C. The first cycle was preceded by 7 min denaturation at 94°C while the last cycle was followed by a 7 min elongation step at 72°C. PCR for human β-globin gene was performed to establish the presence of amplifiable DNA and to exclude the presence of inhibitory factors of the PCR reaction [24]. Sequencing of the GP5+/GP6+ amplimer was performed as follows. PCR reaction was slightly modified, using 1 pM of each primer, 1.5 mM MgCl2 and 1.25 units of AmpliTaq Gold (Applied Biosystem, Foster City, CA). Amplification conditions were as previously indicated. PCR products were purified using the NucleoSpin^® ^Extract II kit (Macherey-Nagel, Düren-Germany) according to manufacturer's instruction. DNA sequencing was performed by Eurofins MWG Operon/M-Medical (Milano, IT). Sequencing results were verified in our laboratory in both sense and anti-sense directions using DNA STAR PC software (Lasergene, Madison, WI USA). HPV type was determined using the BLAST (Basic Local Alignment Search Tool) Search function of the Papilloma Virus Episteme (PaVE) Database (http://pave.niaid.nih.gov).

The patient was a 45-year-old woman who had been referred to our Gynaecology Department because of persistent abnormal vaginal bleeding. Gynaecological examination revealed diffuse enlargement of the cervix which had been replaced by an exophytic ulcerated, reddish lesion. Transvaginal ultrasonography showed slight endometrial irregularities in the whole uterine cavity suggestive of endometrial neoplasms. Pelvic magnetic resonance imaging revealed diffuse enlargement of the cervix which had been replaced by a mass. Induration extended to the parametria and sigmoid colon fat. (Preoperative FIGO stage: IIB).

**Figure 1:**
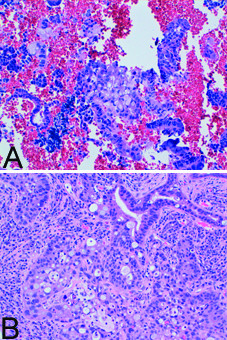
**On histological examination both endometrial curettage (A: Haematoxylin-Eosin × 100) and cervical biopsy (B: Haematoxylin-Eosin × 200) specimens revealed the presence of a neoplasm characterized by neoplastic signet-ring cells and trabecular structures**.

Endometrial curettage and cervical biopsy were performed. On histological examination, both specimens revealed the presence of a neoplasm characterized by neoplastic signet-ring cells and trabecular structures (Figure 1).

Periodic acid Schiff (PAS) and blue alcian stains revealed the presence of intracellular mucin in the signet-ring cells.

**Figure 2:**
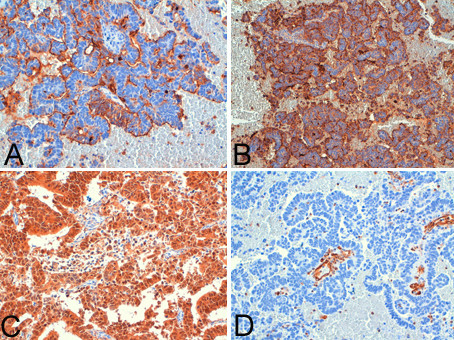
**Immunohistochemical analysis of endometrial neoplasm showed findings consistent with a primary cervical neoplasm, i.e. positivity to Ca-125 (A × 200), CEA (B × 100), and p16 (C × 200) and negativity to Vimentin (D × 200)**.

**Figure 3:**
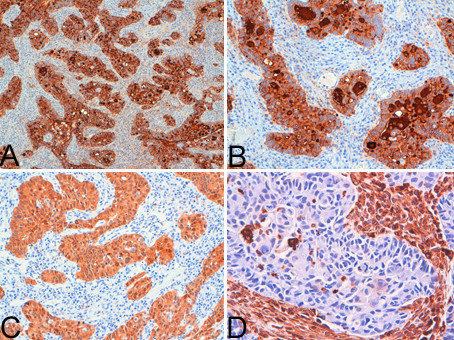
**Immunohistochemical analysis of cervical neoplasm showed findings consistent with a primary cervical neoplasm, i.e. positivity to Ca-125 (A × 100), CEA (B × 200), p16 (C × 200) and negativity to Vimentin (D × 400)**.

**Figure 4:**
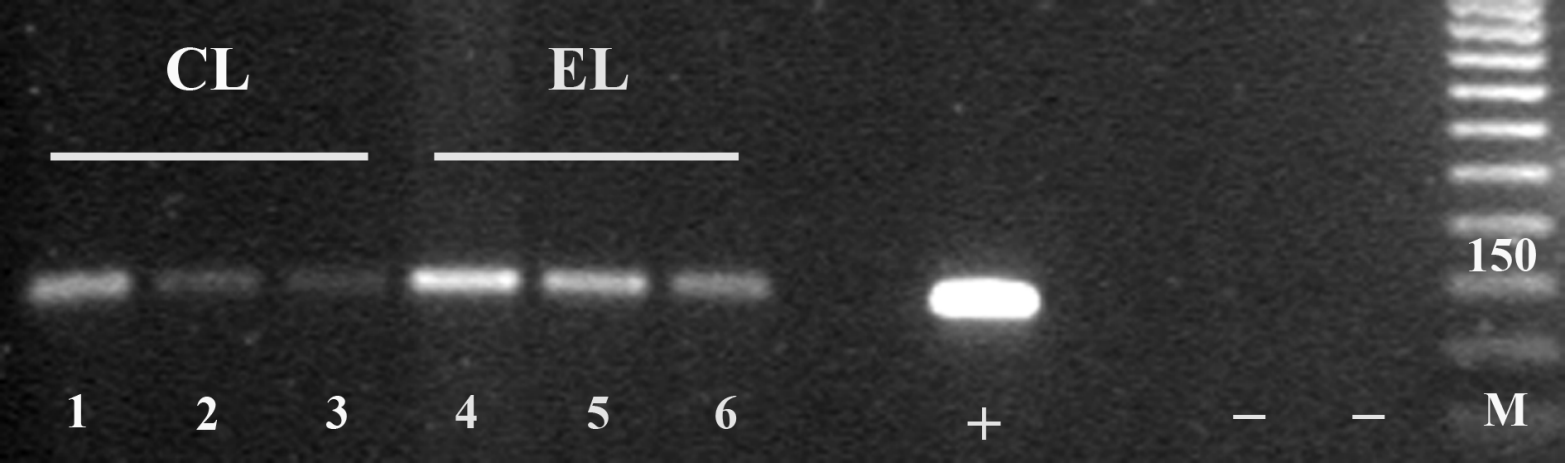
**Results of HPV-PCR amplification using the L1 consensus primers Gp5+/Gp6+ showing strong positivity for HPV-DNA in both endometrial and biopsy specimens: *Lanes 1, 2, 3*: CL (Cervical neoplastic lesion) DNA at three different dilutions, with strong signal for HPV-DNA**. *Lanes 4:, 5, 6: *EL (Endometrial neoplastic lesion) DNA at three different dilutions, with strong signal for HPV-DNA. *M: *molecular weight standard; **+ **= HPV positive control; **- **= HPV negative control.

**Figure 5:**
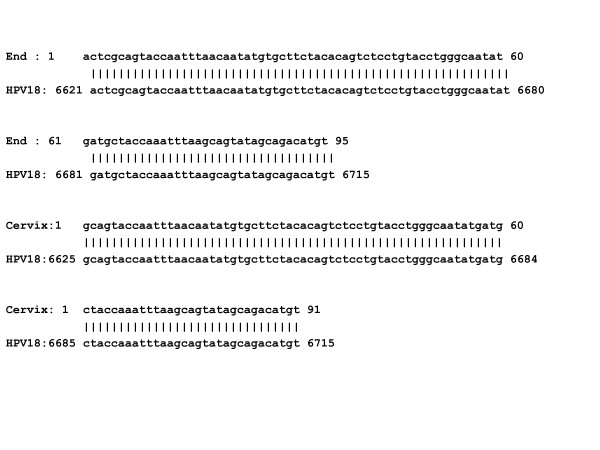
**Sequence alignment illustrating 100% identities between Human papillomavirus type 18 reference sequence (PAVE entry X05015.1) and HPV DNA from both endometrial and cervical specimens**. (End: endometrial lesion)

Immunohistochemical analysis showed findings consistent with a primary cervical neoplasm, including positivity to keratin 7, Ca-125, CEA, and p16 and negativity to Vimentin in both endometrial (Figure 2) and cervical biopsy (Figure 3), oestrogen and progesterone receptors. The cervical origin of the neoplasm was also supported by the presence of HPV DNA (Figure 4) by Polymerase chain reaction amplification (PCR) and sequencing analysis which demonstrated the presence of HPV 18 (Figure 5).

The cervical origin of the neoplasm was further demonstrated by the absence of other neoplasms on oral endoscopy, thorax, abdominopelvic computed tomography, colonoscopy and mammography study.

The absence of immunoreactivity to Chromogranin A and synaptophysin excluded neuro-endocrine differentiation.

A total abdominal hysterectomy, bilateral salpingo-oophorectomy, and selective pelvic lymphadenectomy, omentectomy and resection of sigmoid colon were performed.

Macroscopic examination of the hysterectomy specimen revealed an ulcerated cervical mass and irregularities in the whole endometrial cavity. On cut section, white neoplastic tissue extended through the entire myometrial wall thickness to involve the uterine serosa. Examination of bilateral salpingo-oophorectomy specimens revealed a right ovarian cystic lesion with multiple white nodules on its surface. White neoplastic nodules were also observed in the sigmoid colon fat.

Microscopic evaluation confirmed the initial diagnosis of primary cervical adenocarcinoma with signet-ring elements and prominent endometrial and myometrial involvement.

**Figure 6:**
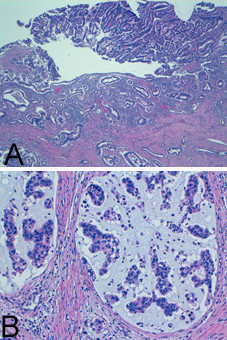
**Microscopic examination of hysterectomy specimen revealed the presence of neoplasm with endometrial and myometrial involvement**. The endometrial component of the neoplasm was formed by predominant trabecular and glandular structures (A: Haematoxylin-Eosin × 40) In the myometrial part, the tumour was characterized by lakes of mucus in which neoplastic elements floated (B: Haematoxylin-Eosin × 100).

Characteristically, the entire endometrial mucosa and myometrial wall thickness were involved. The endometrial component consisted of trabecular and glandular structures and signet-ring elements which accounted for 30% of the tumour (Figure 6A). In the myometrial part, the tumour was characterized by lakes of mucus in which neoplastic elements were floating (Figure 6B). All the nodules observed in the sigmoid colon fat and the cystic lesion of the right ovary were metastases of the cervical neoplasm, while other metastases were observed in 3 pelvic lymph nodes.

## Discussion

It is universally recognized that adenocarcinomas which accumulate mucin in large single or aggregated intracytoplasmic vacuoles displacing the nucleus peripherally, correspond to signet-ring cell carcinoma.

In certain tissues these malignancies are generally characterized by poor prognosis [25,26].

Several researchers have reported artifactual vacuoles and non-adenocarcinomatous neoplasms in various organs and tissues, including the prostate, lymphomas and the ovaries [27-30].

Non-neoplastic endometrial stromal signet-ring cells may be observed during decidualization, when endometrial stromal cells acquire cytoplasmic vacuoles secondary to the accumulation of glycogen and glycoproteins [31,32]. A lack of staining for mucin as well as immunohistochemical non-reactivity for epithelial markers typically allows recognition of these non-neoplastic elements.

The presence of signet-ring cells in a carcinoma within the cervix or in other organs of the genital tract strongly suggests the possibility of a metastasis from a primary tumour of the breast or gastrointestinal tract [3,6-9]. Immunohistochemical and molecular studies have provided information for differential diagnosis in some instances. Monteagudo *et al *observed that in the majority of cases, the signet-ring variant of breast cancer metastatic to the ovary showed positivity to gross cystic disease fluid protein -15 (GCDFP-15) [33]. In contrast, immunoreactivity to colorectal antigens such as CEA, CDX-2 (20) and CK 20 [19] in a cervical lesion does not rule out a diagnosis of primary cervical carcinoma.

As well as cervical cases, adenocarcinomas express markers common to gastric, intestinal and pancreatobiliary epithelial cells [34]. Moreover, simultaneous positivity to CEA and keratin 7 do not differentiate between PCSRCC and mammary metastatic malignancy [35].

To the best of our knowledge, only 14 cases of primary cervical carcinoma containing signet-ring cells have been reported in the English literature [13-22] (Table 1).

**Table 1:** Previous Reported cases of PCSRCC

*Authors*	*Age (yrs)*	*Presenting symptoms*	*Histological features associated* *and* *FIGO stage*	*HPV*	*ER/PGR*	*Treatment *	*Outcome*
Moll et al (1990)	50	Post-coital vaginal bleeding, menometrorrhagia	III (metastases of ovary and lymph nodes)	Not done	Not done	Surgery and whole pelvic irradiation	DOD 10 mo

Mayorga et al (1997)	68 74	Post-coital bleeding Post-menopausal bleeding	Ib Ib	Not done Not done	Not done Not done	Preoperative chemotherapy and surgery Surgery	NED 35 mo NED 25 mo

Haswani et al (1998)	33 38	Asymptomatic AGC-NOS on a routine vaginal smear) Post-coital vaginal bleeding In both cases previous cervical condylomas	Adenosquamous ca, III, N1 AIS/HSIL Ib N1	HPV type 18 by PCR Not done	-/ --/-	Palliative radio and chemotherapy Surgery and radiotherapy	DOD 10 mo NED 18 mo

Cardosi et al. (1999)	53	Perimenopausal bleeding	neuroendocrine differentiation Ib	Not done	+/+	Surgery, radiotherapy Chemotherapy	NED 6 mo

Moritani et al (2004)	29	Persistent abnormal genital bleeding	glassy cell component III b N1	- by immunohistochemistry	-/-	Chemotherapy	NED 6 mo

Insabato et al (2007)	46	Vaginal bleeding in cervical polypoid lesion	Ib	Not done	Not Done	Surgery radiotherapy Chemotherapy	NED 3 yrs

Suárez-Peñaranda (2007)	80	Vaginal discharge	neuroendocrine differentiation IIIb	Not done	Not Done	Radiotherapy/Chemotherapy	DOD 18 mo

McCluggage WG et al (2008)	NR	Two cases NR	Foci of signet-ring cell in the usual type of cervical adenocarcinoma Diffuse positivity to P16	Not done	Not Done	NR	NR

Versas E et al (2009)	36 43	Thromboembolic events (Trousseau Syndrome)Metastases of lung and lymph nodes		+ HPV by in situ hybridization	-/- -/-	Chemotherapy Chemotherapy	DOD 7 wks DOD 2 mo

Balci et al (2010)	53	Post-menopausal bleeding	(0% of neoplasms with SRC II b3x 5 × 2 parametrio N1	HPV type 18	-/-	Surgery	NR

AGC-NOS: Atypical Glandular Cells not otherwise specified

AIS: adenocarcinoma in situ

DOD: dead of disease

HSIL: High-grade Squamous Intraepithelial Lesion

HPV: human papilloma virus

Mo: months NED: not evidence of disease

NR: not reported

PCR: Polymerase chain reaction amplification

Yrs: years

Wks: weeks

The majority of the patients in these reports are postmenopausal women [13,14,16,19,22]. Conspicuous signet-ring cells were reported in 7 cases [13,15,16,18,19,22], while concomitant glandular structures [16,20] or tubular and tubuloglandular formations [13,15] have been described in some cases. In the case reported by Insabato *et al*, the neoplasm was associated with *adenocarcinoma in situ *(AIS) [18] and in that described by Haswani *et al*, both AIS and a high-grade squamous intraepithelial lesion were present [15], but these were absent in our case. A simultaneous glassy cell component was observed in a young Japanese woman by Moritani *et al *[17], while partial enteric immunophenotype with consistent expression of CDX2 marker has been described by McCluggage *et al *[20].

Oestrogen and Progesterone receptors have been tested in only 4 previous cases [15-17,19,21] and these were present only in one example [16].

Neuroendocrine differentiation by using neuroendocrine markers such as Chromogranin A [19] and both Chromogranin A and Synatophysin [16] have been demonstrated in two cases, but these markers were negative in our case.

In our case, as well as in some previous published examples of the PCSRCC, the primary cervical origin was supported by the presence of HPV DNA using molecular analysis [15,22] and by P16 immunoreactivity [20,22], which may be considered a surrogate marker for HPV infection [36].

Prognosis for PCSRCC is not well understood because of its rarity [13-22]. Moreover, is not possible to draw convincing conclusions about the prognostic significance of signet-ring component in endocervical carcinoma since the follow-up period in the majority of the previous cases reported in the literature is either too short [15-17] or was not reported [20,22] (Table 1). However, in our opinion, prognosis in this rare malignancy should be considered poor at more advanced stages of the disease.

This hypothesis is supported by data in the literature which show that all patients with an advanced stage of development of neoplasm (stage III sec FIGO) died from the disease after only 10 [13] or 18 months [15,19] and 7 weeks and 2 months from diagnosis [21]. Moreover, in these examples of PRSCC, the authors had also documented that this rare malignancy showed resistance to radiotherapy and/or chemotherapy [13,15,19,21]. In cases observed by Veras et al, the PCSRCC were characterized by widespread metastatic disease with systemic thromboembolic phenomena (Trousseau syndrome) and both patients expired shortly after initiation of chemotherapy (Table 1). In these cases, Veras et al excluded metastatic adenocarcinomas of the upper gastrointestinal tract origin because of the presence of HPV DNA by in situ hybridization [21].

In cases with a lower stage of development (stage Ib sec FIGO) prognosis is better. Indeed, in the cases described by Mayorga *et al *and Insabato *et al*, the patients showed no evidence of disease several months after diagnosis [14,18].

Generally, cervical carcinoma spreads to the parametria and/or vagina. In our case, the neoplasm had involved the uterine corpus and showed prominent endometrial and myometrial infiltration simulating primary endometrial adenocarcinoma with cervical extension. Data from the literature reveal that the spread of cervical carcinoma to the uterine corpus, although rare, may be observed [16,37-39].

As in our case, in the series of Yemelyanova *et al*, the cervical origin of the malignancy was demonstrated using immunohistochemical and molecular studies which respectively revealed immunoreactivity to p16 and negativity to hormone receptors plus the presence of HPV DNA in both the corpus and cervical component of the neoplasm [39].

In our case, negativity to Vimentin and positivity to CEA proved useful in excluding primary signet-ring cell carcinoma of the endometrium an entity even rarer than PCSRCC [10-12].

Positivity to p16 protein and molecular analysis using PCR revealed that this rare entity is related to HPV infection in the same way as other subtypes of primary adenocarcinoma [39-42].

## Conclusion

With this report the authors have demonstrated that the spread of cervical adenocarcinoma to the uterine corpus, although rare, may be observed, and that in this instance immunohistochemical and molecular studies can provide sufficient information for accurate diagnosis even on small biopsy specimens.

### Consent

Written informed consent was obtained from the patient for publication of this case report and any accompanying images. A copy of the written consent is available for review by the Editor-in-Chief of this journal.

## Abbreviations

AGC-NOS: Atypical Glandular Cells not otherwise specified; AIS: adenocarcinoma in situ; BLAST: Basic Local Alignment Search Tool; CL: Cervical neoplastic lesion; DOD: Dead Of Disease;EL: Endometrial neoplastic lesion; End : endometrial lesion; GCDFP-15: Gross Cystic Disease Fluid Protein -15; HSIL: High-grade Squamous Intraepithelial Lesion; HPV: Human Papilloma Virus; M: molecular weight standard; Mo: months; NED: not evidence of disease; NR: not reported; PAS: Periodic acid Schiff; PaVE: Papilloma Virus Episteme; PCR: Polymerase chain reaction amplification; PSRCC: Primary Signet-Ring Cell carcinoma of the Cervix; Yrs: years; Wks: weeks.

## Competing interests

The authors declare that they have no competing interests.

## Authors' contributions

GG: carried out the study design and writing. SP and TD: did molecular study and participated in the literature search. RB: performed the operation of the patient. All authors read and approved the final manuscript.
